# Electronic-Cigarette Vehicles and Flavoring Affect Lung Function and Immune Responses in a Murine Model

**DOI:** 10.3390/ijms21176022

**Published:** 2020-08-21

**Authors:** Brittany N. Szafran, Rakeysha Pinkston, Zakia Perveen, Matthew K. Ross, Timothy Morgan, Daniel B. Paulsen, Arthur L. Penn, Barbara L. F. Kaplan, Alexandra Noël

**Affiliations:** 1 Center for Environmental Health Sciences, Department of Basic Sciences, Mississippi State University College of Veterinary Medicine, Mississippi State, MS 39762, USA; bns267@msstate.edu (B.N.S.); MRoss@cvm.msstate.edu (M.K.R.); bkaplan@cvm.msstate.edu (B.L.F.K.); 2 Department of Comparative Biomedical Sciences, School of Veterinary Medicine, Louisiana State University, Baton Rouge, LA 70803, USA; rpinks2@lsu.edu (R.P.); zperve1@lsu.edu (Z.P.); apenn1@lsu.edu (A.L.P.); 3 Department of Environmental Toxicology, Southern University, Baton Rouge, LA 70803, USA; 4 Department of Pathobiology and Population Medicine, Mississippi State University College of Veterinary Medicine, Mississippi State, MS 39762, USA; Morgan@cvm.msstate.edu; 5 Pathobiological Sciences, School of Veterinary Medicine, Louisiana State University, Baton Rouge, LA 70803, USA; dpauls1@lsu.edu

**Keywords:** electronic nicotine delivery systems—electronic-cigarette, aberrant lipidomics, lung immune homeostasis, vanilla flavoring, vegetable glycerin—propylene glycol

## Abstract

The use of electronic nicotine delivery systems (ENDS), also known as electronic-cigarettes (e-cigs), has raised serious public health concerns, especially in light of the 2019 outbreak of e-cig or vaping product use-associated acute lung injury (EVALI). While these cases have mostly been linked to ENDS that contain vitamin E acetate, there is limited research that has focused on the chronic pulmonary effects of the delivery vehicles (i.e., without nicotine and flavoring). Thus, we investigated lung function and immune responses in a mouse model following exposure to the nearly ubiquitous e-cig delivery vehicles, vegetable glycerin (VG) and propylene glycol (PG), used with a specific 70%/30% ratio, with or without vanilla flavoring. We hypothesized that mice exposed sub-acutely to these e-cig aerosols would exhibit lung inflammation and altered lung function. Adult female C57BL/6 mice (*n* = 11–12 per group) were exposed to filtered air, 70%/30% VG/PG, or 70%/30% VG/PG with a French vanilla flavoring for 2 h a day for 6 weeks. Prior to sacrifice, lung function was assessed. At sacrifice, broncho-alveolar lavage fluid and lung tissue were collected for lipid mediator analysis, flow cytometry, histopathology, and gene expression analyses. Exposures to VG/PG + vanilla e-cig aerosol increased lung tidal and minute volumes and tissue damping. Immunophenotyping of lung immune cells revealed an increased number of dendritic cells, CD4+ T cells, and CD19+ B cells in the VG/PG-exposed group compared to air, irrespective of the presence of vanilla flavoring. Quantification of bioactive lung lipids demonstrated a >3-fold increase of 2-arachidonoylglycerol (2-AG), an anti-inflammatory mediator, and a 2-fold increase of 12-hydroxyeicosatetraenoic acid (12-HETE), another inflammatory mediator, following VG/PG exposure, with or without vanilla flavoring. This suggests that e-cig aerosol vehicles may affect immunoregulatory molecules. We also found that the two e-cig aerosols dysregulated the expression of lung genes. Ingenuity Pathway Analysis revealed that the gene networks that are dysregulated by the VG/PG e-cig aerosol are associated with metabolism of cellular proteins and lipids. Overall, our findings demonstrate that VG and PG, the main constituents of e-liquid formulations, when aerosolized through an e-cig device, are not harmless to the lungs, since they disrupt immune homeostasis.

## 1. Introduction

In the past year, the general public’s perception regarding the safe use of electronic nicotine delivery systems (ENDS) was challenged with the 2019–2020 electronic-cigarette (e-cig) or vaping product use-associated acute lung injury (EVALI) outbreak, throughout the United States. As of the end of February 2020, the Centers for Disease Control and Prevention (CDC) had confirmed over 65 deaths and more than 2800 cases of EVALI. The CDC and Food and Drug Administration (FDA) are currently conducting work to determine the cause of this recent outbreak, but the majority of the e-liquid samples tested were found to contain Vitamin E acetate, a thickening agent used in ENDS containing delta-9-tetrahydrocannabinol (THC) [1,2]. EVALI, however, has been diagnosed in patients since 2015 [3,4,5,6], and is still observed in nicotine-exclusive ENDS users [7]. While EVALI cases have all been acute, there is limited research on the long-term safety of e-cig use, particularly regarding the use of e-cig delivery vehicles [8,9]. ENDS e-liquid typically contains a combination of delivery solvents, including vegetable glycerin (VG) and propylene glycol (PG), plus nicotine, flavorings, and other additives [3]. In recent years, over 400 different brands of ENDS devices and more than 7000 flavors have been identified, with increasing numbers monthly [10]. This means the composition of ENDS varies widely and can be difficult and complex to study. The addition of flavoring has made ENDS appealing to teenagers, who perceive flavored ENDS as more enjoyable to use than unflavored ENDS [11], and thus, use in this age group has increased tremendously from 1.5% in 2011 to 20.8% in 2018 [12]. ENDS were originally marketed as a safer alternative to traditional cigarettes, but approximately 11% of adult ENDS users and 40% of young adult ENDS-users were originally non-smokers [10]. Since little is known regarding the long-term health effects of vaping, ENDS pose a significant public health concern and should be evaluated further for their safety.

Scientific evidence regarding the safety of ENDS is still a matter of debate. A limited number of studies have assessed whether ENDS cause lung inflammation. At least one study has found no difference in lung cytokine levels 4 months after ENDS exposure, with or without nicotine [9]. This same study, however, found differences in inflammation and immune responses after influenza challenge [9]. In contrast, several studies have identified increases in cytokines associated with ENDS use, including IL-6 and IL-8, in both in vivo and in vitro studies [13,14,15,16,17]. Flavorings, with or without nicotine, have been reported to produce varying effects on lung inflammation, depending on the specific flavoring [18].

A recent study demonstrated that mice exposed to e-cig aerosols composed solely of delivery vehicles (PG and VG), without nicotine, responded with aberrant lipidomics [9]. This study found an increase in lipid accumulation in alveolar macrophages that was due to increases in phospholipid species that were linked to increases in surfactant-associated lipid species and decreases in gene expression related to lung SP-A and SP-D [9]. Other studies have also identified atypical macrophage lipid accumulation and aberrant lipid levels [4,19,20,21,22]. Pulmonary surfactant proteins (SP) are composed of 90% lipids and function to increase pulmonary compliance, facilitate immune defenses (SP-A and SP-D), and regulate surfactant phospholipid metabolism [23]. In addition to the Madison et al. [9] study, other studies have also evaluated the effects of ENDS on pulmonary surfactant proteins. These in vitro studies found no differences in function at doses consistent with typical ENDS use although structure of the proteins was altered [24,25]. As multiple studies have identified a disruption in lipid homeostasis and pulmonary surfactant proteins due to ENDS exposure with and without nicotine, further investigation into these findings, particularly with lipid-based immune mediators, is warranted.

Fruit, dessert and candy, are currently the trendiest U.S. e-liquid flavor categories [26]. Ethyl vanillin and vanillin are characteristic aldehyde flavoring chemicals that provide the vanilla flavor for many desert flavored e-liquids, including vanilla cream, vanilla butternut, crème brûlée, custard, cheesecake, and cream pie [27]. In the present study, we used a murine model to study the sub-acute pulmonary effects of inhaling e-cig delivery vehicles containing 70%/30% VG/PG with or without vanilla flavoring. We chose to investigate the effects of French vanilla flavored e-liquid, since it is composed of widely-used flavoring chemicals (vanillin and ethyl vanillin) in popular American e-liquids. In addition, vanilla flavoring has the potential to inhibit the activity of monoamine oxidase (MAO) enzymes, and thus may have pharmacological/toxicological effects [28]. Both VG and PG are classified “generally recognized as safe” (GRAS) by the FDA as food additives, but as substances inhaled through an ENDS device, their safety has not yet been thoroughly assessed. Heating and aerosolizing humectants (PG and VG) via an e-cig device allows for the formation of reactive aldehyde species, including acetaldehyde and acrolein, which are known to have significant harm potential for the lungs [29,30]. The pulmonary toxicity of these aldehydes can be added to the toxicity of the parent PG component, which is considered an upper airway irritant, and has been associated with an increased risk for asthma [31,32,33]. Besides, higher VG ratios in ENDS allow for a smoother vaping experience and produce high aerosol levels [31]. Unlike many other studies that focus on a higher concentration of PG/VG, our study focuses on a higher VG/PG ratio. These VG skewed ratio e-liquids are popular with a growing subclass of youth and young adult e-cig users, the “cloud chasers”, who pride themselves on the size of the exhaled aerosol cloud that the skewed VG/PG ratios enable. Importantly, varying the ratios of PG/VG in e-liquid formulation will cause a concomitant change in the levels of exposure to specific harmful aldehydes produced in the e-cig aerosol [31,32,33]. Moreover, the production of “flavor aldehyde—solvent acetal” will occur in e-liquids composed of aldehyde flavoring chemicals, including ethyl vanillin and vanillin [34]. The carryover of the acetals from the e-liquid to the e-cig aerosol is substantial—at 50%–80%—which could lead, following inhalation, to respiratory tract irritation [27,34]. Vanillin VG acetal and vanillin PG acetal are produced in vanillin containing e-liquids and the vanillin PG acetal has a greater pro-inflammatory effect than vanillin [27,34]. Furthermore, the quantity of acetal present in both e-liquids and e-cig aerosols is dependent upon the e-liquid PG/VG ratio [27]. To our knowledge, no studies have investigated the effects of inhaled vanilla-flavored e-cig aerosol, including the potential for the formation of harmful aldehydes plus vanillin PG and VG acetals, on lung function and immune responses. The goals of this study were to (i) determine the effect of one particular ratio of delivery vehicles, 70% VG/30% PG, on lung function, (ii) evaluate markers of lung inflammation associated with this ratio of delivery vehicles, and (iii) determine the effects of those delivery vehicles on inflammatory lipid mediators, with or without vanilla flavoring. We hypothesized that sub-acute exposures to 70% VG/30% PG will disrupt lung function, increase lung tissue macrophages and other immune cell types, increase markers of inflammation, and disrupt normal lung immune lipid mediators.

## 2. Results

### 2.1. VG/PG Plus Vanilla Impaired Lung Functional Parameters

**Figure 1:**
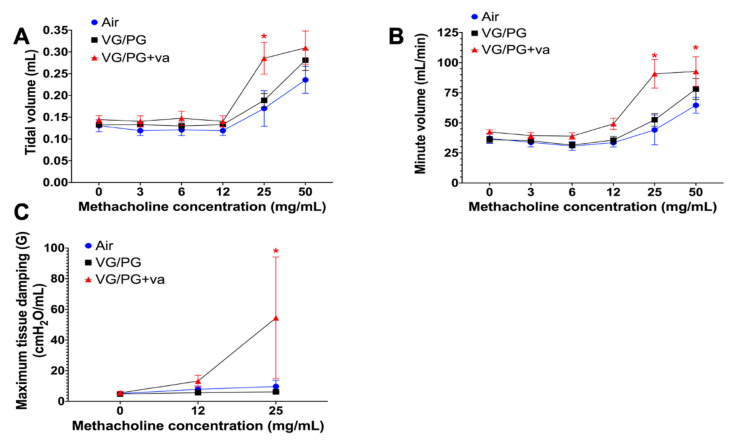
Inhalation of e-cig aerosol composed of 70%/30% VG/PG plus vanilla flavor impairs lung function. Whole-body plethysmography with methacholine challenge was used to determine tidal volume (**A**), and minute volume (**B**) (*n* = 8 per group), while the flexiVent^®^ system was used to assess the maximum tissue damping (**C**) (*n* = 6 per group). Data are expressed as mean ± standard error of the mean (SEM). Differences were analyzed by a two-way ANOVA. * *p* < 0.05.

Results of whole-body plethysmography and flexiVent lung function testing with methacholine challenge did not differ between air controls and VG/PG (Figure 1). At methacholine doses greater than 25 mg/mL, the VG/PG plus vanilla group demonstrated greater tidal and minute volumes than the air control group (*p* < 0.05) (Figure 1A,B). Tissue damping (Figure 1C), measured via the flexiVent, was also significantly greater in the VG/PG plus vanilla group at 25 mg/mL methacholine (*p* < 0.05) compared to the VG/PG and the air control groups. The other lung function parameters measured via the flexiVent system were not significantly changed (data not shown).

### 2.2. VG/PG and VG/PG Plus Vanilla Did not Alter Lung Macrophage Counts

**Figure 2:**
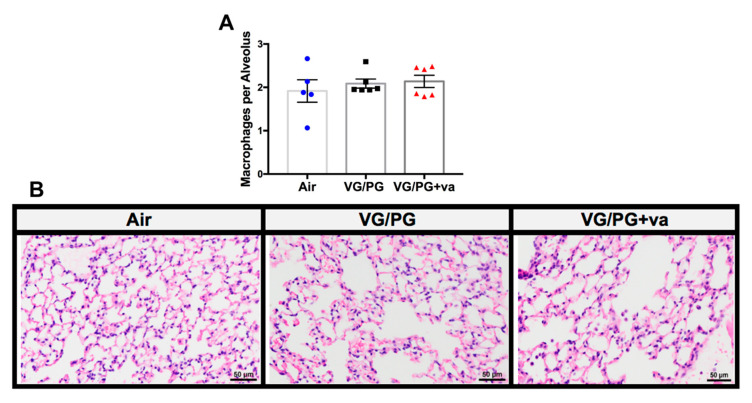
Inhalation of e-cig aerosol composed of 70/30% VG/PG alone or with vanilla flavor does not impact lung tissue macrophages. H&E stained lung slides from mice treated with air (*n* = 5), VG/PG (*n* = 6), and VG/PG plus vanilla (*n* = 6) were evaluated for the number of macrophages in 5 representative images at 40x. (**A**) Counts were normalized to the number of alveoli per image to account for differences in lung section inflation. (**B**) Representative images of H&E stained slides of mouse lungs at 400× magnification.

Macrophage counts on hematoxylin and eosin (H&E) stained lung slides from mice treated with VG/PG and VG/PG plus vanilla were not significantly different from the air control values (Figure 2). Counts were normalized to the number of alveoli per image due to differences in inflation of the lungs. In addition, globally the lung tissue from the air controls and the two exposure groups of mice appeared normal, with no significant changes in lung histopathology. Rarely, eosinophils were observed in the lung tissue of VG/PG− and VG/PG plus vanilla-exposed mice. The histology of the nasal passages of all mice was also unremarkable.

### 2.3. VG/PG Altered Lung Cell Immunophenotype

**Figure 3:**
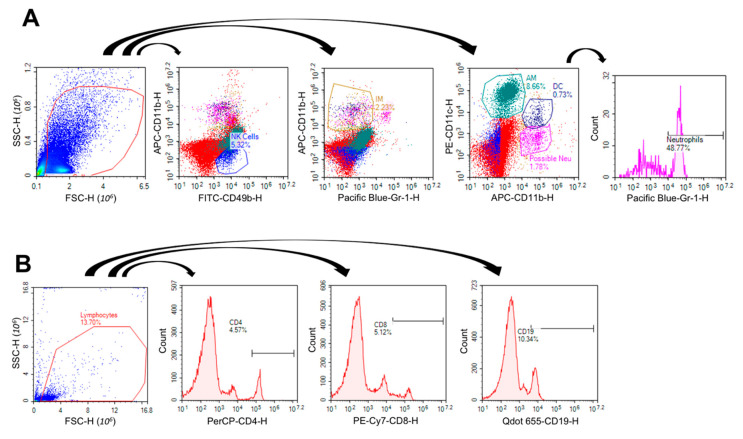

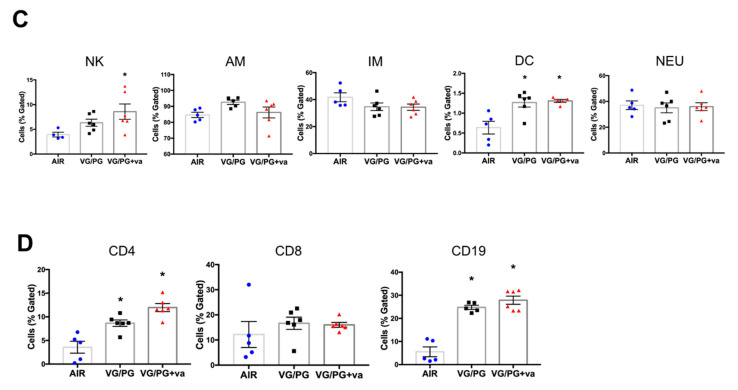
Inhalation of e-cig aerosols composed of 70%/30% VG/PG alone or with vanilla flavor increases lung immune cells. (**A**,**B**) Gating Strategy for Identifying Lung Innate and Adaptive Immune Cells. (**A**) NK cells were identified as CD49+/CDllb−, AMs as F4/80+/CD11c+/CD11b−, IMs as F4/80+/CD11b+/Gr1−, DCs as CD11b+/CD11c+, and Neutrophils as CD11b+/CD11c−/Gr1+. (**B**) T helper cells were identified as CD4+, cytotoxic T cells as CD8+, and B cells as CD19+. Extracellular staining for innate (**C**) and adaptive (**D**) immune cells in the lungs of mice treated with air (*n* = 5), VG/PG (*n* = 6), or VG/PG (*n* = 6) with vanilla. * *p* < 0.05. Cells were identified as natural killer cells (NK, CD49+, CDllb−), alveolar macrophages (AM, F4/80+, CD11c+, CDllb−), interstitial macrophages (IM, F4/80+, CDllb+, Gr1−), dendritic cells (DC, CDllb+, CDllc+), neutrophils (NEU, CD11b+, CDllc−, Gr1+), cytotoxic T cells (CD8+), T helper cells (CD4+), or B cells (CD19+). Error bars are SEM. Differences were analyzed by one-way ANOVA, and a Grubb’s outlier test was utilized to identify outliers in each treatment group.

The gating strategy for identifying lung innate and adaptive immune cells is shown in Figure 3A,B. The VG/PG and VG/PG plus vanilla samples did not produce any significantly different results compared to air controls for alveolar macrophages (F4/80+, CD11c+, CD11b−), interstitial macrophages (F4/80+, CD11b+, Gr1−), or neutrophils (CD11b+, CD11c−, Gr1+) (Figure 3C). VG/PG alone did not significantly alter the percentage NK cells, but there was a slight significant increase in VG/PG plus vanilla compared to air controls (*p* = 0.0214). Both VG/PG and VG/PG plus vanilla groups displayed significantly increased dendritic cell populations (CD11b+, CD11c+) versus air controls (*p* = 0.0161 & *p* = 0.0142, respectively). Regarding the adaptive immune cell responses, VG/PG and VG/PG plus vanilla did not alter cytotoxic T cell populations (CD8+), but compared to controls, VG/PG significantly increased both T helper cell (CD4+, *p* = 0.0280 & *p* = 0.0077, respectively) and B cell populations (CD19+, *p* = 0.0003 & *p* = 0.0001, respectively), regardless of whether vanilla flavoring was present (Figure 3D).

### 2.4. VG/PG Increased Levels of Lipid Mediators

**Figure 4:**
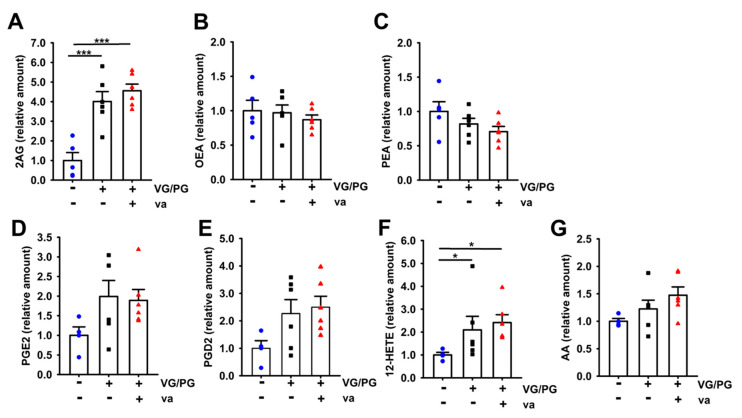
Inhalation of e-cig aerosols composed of 70%/30% VG/PG alone or with vanilla flavor affects the extracted amount of endocannabinoid and prostaglandins in the lungs. The right middle lung lobe was extracted for lipid mediators and quantified by LC-MS/MS using deuterated standards for mice exposed to air (*n* = 5), VG/PG (*n* = 6), and VG/PG (*n* = 6) plus vanilla. 2-AG (**A**), OEA (**B**), PEA (**C**), PGE2 (**D**), PGD2 (**E**), 12-HETE (**F**), and AA (**G**) levels are pictured. AEA levels were too low to quantify. Data are expressed as mean ± SEM. Differences were analyzed by a one-way ANOVA (* *p* < 0.05, *** *p* < 0.001).

We assessed the levels of lipid mediators that have immunomodulatory effects, either through their pro- or anti-inflammatory properties. Exposures to VG/PG and VG/PG plus vanilla significantly increased the pulmonary levels of the endocannabinoid 2-AG (*p* < 0.001) by approximately four-fold over air controls (Figure 4A). Neither of those exposures, however, altered the pulmonary levels of OEA (Figure 4B) or PEA (Figure 4C). AEA levels were not quantifiable by our method. Additionally, VG/PG and VG/PG plus vanilla significantly increased levels of 12-hydroxyeicosatetraenoic acid (12-HETE), approximately two-fold over air controls (Figure 4F, *p* = 0.043 & *p* = 0.019, respectively). Although the values did not reach statistical significance, there was an increased trend in PGE2 (Figure 4D), PGD2 (Figure 4E), and AA (Figure 4G) levels in the VG/PG and VG/PG plus vanilla groups over the air control group.

### 2.5. Alteration of Gene Expression by VG/PG and VG/PG plus Vanilla

**Figure 5:**
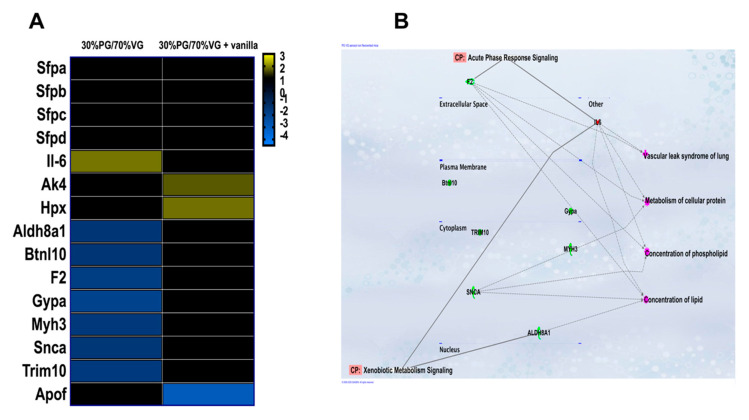
Inhalation of e-cig aerosols composed of 70%/30% VG/PG alone or with vanilla flavor dysregulates lung gene expression. (**A**) Heatmap demonstrating the expression of immunotoxicity related genes in lungs of mice. Black color denotes no difference in fold-change compared to air controls. (**B**) Ingenuity Pathway Analysis (IPA) gene networks associated canonical pathways and function. Data are expressed in fold-changes compared to air control group (*n* = 4–6 mice per group). A greater than ±1.5-fold change is considered significant.

Several genes were dysregulated in response to VG/PG or VG/PG plus vanilla (Figure 5). One gene, *Il-6*, was upregulated and seven genes, *Aldh8a1*, *Btnl10*, *F2*, *Gypa*, *Myh3*, *Snca*, and *Trim10*, were downregulated in response to VG/PG alone. Two genes, *Ak4* and *Hpx*, were upregulated, and one gene, *Apof*, was downregulated in response to VG/PG plus vanilla. The PG/VG e-cig aerosol dysregulated genes related to biotransformation (*Aldh8a1*), transcription factors expressed in pulmonary surfactant (*F2*) [35], synuclein-alpha (*Snca*), which interacts with phospholipids and proteins, as well as *Il-6*, a multifunctional cytokine, whose enhanced expression was correlated with reduced production of pulmonary surfactant protein A (SPA) [36]. The VG/PG plus vanilla e-cig aerosol increased the expression of *Hpx*, which protects against oxidative damage and affects the anti-inflammatory properties of high-density lipoprotein (HDL) [37]. Ingenuity Pathway Analysis (Figure 5B) revealed that the gene networks that are dysregulated by the PG/VG e-cig aerosol we used are associated with metabolism of cellular proteins and with control of lipid levels.

### 2.6. Immunoglobulin Levels Were Altered by VG/PG plus Vanilla

**Figure 6:**
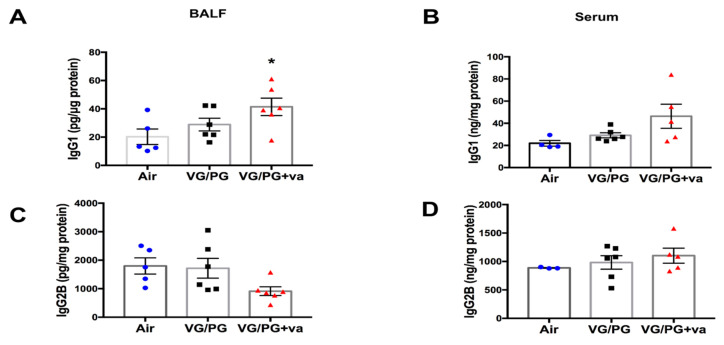
Inhalation of e-cig aerosols composed of 70%/30%VG/PG plus vanilla flavor alters IgG1 levels in bronchoalveolar lavage fluid (BALF). IgG1 and IgG2b levels measured by ELISA (**A**,**C**) in serum and BALF (**B**,**D**) (*n* = 6 in all groups except the air control where *n* = 5). Values are normalized on protein. * *p* < 0.05. Error bars are SEM, and differences were analyzed by one-way ANOVA.

Based on the increases in T helper cells and B cell populations, we investigated whether there were corresponding increases in IgG1 and IgG2b antibody production by ELISA in serum and BALF (Figure 6). The levels of IgG1 and IgG2b were not altered in serum or BALF from animals exposed to VG/PG, but there was a significant increase in IgG1 levels in the BALF of animals exposed to VG/PG plus vanilla over air controls (*p* = 0.0431).

## 3. Discussion

PG and/or VG are key constituents of e-liquid formulations and are used in virtually all ENDS devices [4]. These delivery vehicles are heated through an e-cig device at 200 °C or greater [38]. The thermal degradation of VG plus the interaction with the other e-liquid constituents produce emissions of aldehydes, including formaldehyde and acetaldehyde, known to be potent threats to human health [29,30]. PG and VG are biologically relevant molecules with emulsifying properties, which in the form of an e-cig aerosol, can interact with cells of the respiratory tract and potentially impair lipid homeostasis in the lungs [4]. In this study, we examined the effects of a 70/30% VG/PG e-cig aerosol, with or without French vanilla flavoring, on mouse lung function and inflammation. We identified increases in markers associated with lung immunotoxicity (gene expression), alterations in lung cell immunophenotyping, and immunosuppression (lipid-based immune mediators) due to VG/PG exposure.

We used two complementary methods to evaluate lung function in mice, non-invasive whole-body plethysmography, and invasive forced oscillation technique (FOT), enabling us to provide descriptive measures of phenotypic characteristics of lungs that have been exposed sub-acutely to e-cig aerosols. While whole-body plethysmography provides estimates for lung volumes, the FOT allows for accurate measurements of lung mechanics [39]. The most profound effect of adding the vanilla flavoring was on lung function. At a methacholine challenge dose of 25 mg/mL, whole-body plethysmography revealed increased estimates of lung tidal (Figure 1A) and minute (Figure 1B) volumes in mice exposed to the 70%/30% VG/PG plus vanilla e-cig aerosol. This same aerosol also increased G, the maximum tissue damping (Figure 1C), an indicator of lung tissue resistance. It is important to note that they were no significant baseline tissue damping changes (<5 cm H2O/mL) for either e-cig group (Figure 1C). Following bronchoconstriction induced by aerosolized methacholine (25 mg/mL), however, the mice exposed to the e-cig aerosol containing vanilla exhibited increased lung tissue resistance (G) (Figure 1C). Thus, in a context of transient bronchoconstriction, only the mice from the VG/PG plus vanilla group showed a heightened response. Unique responses from this particular group also were manifested in other outcomes, including an increased population of lung NK cells (Figure 3), increased lung gene expression of *Ak4* and *Hpx* (Figure 5), and increased levels of IgG1 in BALF (Figure 6). It was previously reported that increased lung tissue resistance is a multi-factorial event that can include alterations in tissue compliance, viscosity, and cellular infiltration [40]. In addition to the increased percentages of DC, CD4, and CD19 immune lung cells caused by e-cig aerosol exposures, we found an increased NK cell population solely in the VG/PG plus vanilla group (Figure 3). This may be a contributing factor to the impaired lung function observed for this particular group. Indeed, augmented levels of NK cells also are present in BALF from chronic obstructive pulmonary disease (COPD) patients [41]. Further, in mouse cigarette smoke exposure studies, NK cells were linked to chronic airway inflammation [42], as well as being in an enhanced primed and activated state [43]. It is well-known that the COPD phenotype is characterized by decline in lung function [41,42,43]. Overall, these results suggest that changes in immune lung cells, including NK cells, may be related to lung function impairment in a context of exposures to inhaled pollutants. Additionally, the chemical profile of the inhaled pollutants may have an impact on lung function. Ethyl vanillin and vanillin are MAO enzyme inhibitors [28]. Lung MAO functions include the degradation of circulating catecholamines, such as serotonin and norepinephrine [44]. Thus, reduced lung MAO activity can result in an increased concentration of these vasoactive substances, further leading to sympathetic activation [44,45,46], which has been associated with both obstructive, as well as restrictive, lung diseases [45,46]. This suggests that the specific chemical profile of vanilla flavoring may also play a role in the VG/PG plus vanilla group altered lung function.

Lung tissue resistance or damping (G) is a measure that represents tissue physical properties or local heterogeneity in the lungs. G also reflects energy dissipation, which is required for expansion of the lung tissue [39,47,48]. Since we did not observe any significant changes in respiratory system resistance (Rrs) (data not shown), but found an increase in tissue damping (G) at 25 mg/mL of mechacholine (Figure 1C), this suggests that the VG/PG plus vanilla e-cig aerosol exposure caused changes in peripheral lung function rather than in the central airways [39]. This increase in G may be representative of alterations in the peripheral lung, in terms of either inhomogeneity of airflow or increased airway resistance [48], which may be caused by the increased lung cell infiltration (NK, DC, CD4, and CD19) that was observed in the VG/PG plus vanilla group. This pulmonary mechanics phenotype can translate into mice having to spend more energy to expand their lungs [49]. Lung tissue damping (G) is known to increase with increasing lung volumes [50]. This is supported by the estimated increased in tidal and minute volumes in the VG/PG plus vanilla group (Figure 1A,B). It is unknown, however, whether the estimated lung volumes that we measured by whole-body plethysmography are reflective of increased physiological lung volumes, which are associated with obstructive lung diseases and hyperinflation [51,52,53]. Overall, here, in our murine model, we identified an effect of vanilla flavoring on lung function that was not present in the VG/PG alone group. Our results can be compared to the study by Glynos et al. [15] which did not find a difference in murine lung function after a 4 week exposure to PG/VG without flavoring or nicotine. Interestingly, they did see a difference initially after 3 days of exposure with vehicle alone, but no difference with the flavoring (tobacco blend) either at 3 days or 4 weeks [15]. The key here seems to be the flavoring differences. Vanilla flavor affects lung function while tobacco flavor does not. Moreover, in an asthma mouse model, exposures to various unflavored (PG/VG only) and flavored e-cig aerosols, including banana pudding and black licorice, without nicotine, increased the baseline measurements of lung function, including tissue damping (G), following house-dust mite treatment [18]. Impaired lung function was also observed in mice following an 8 week exposure to e-cig aerosol composed of tobacco flavor plus 100% VG or PG, with and without nicotine [54]. This study specifically showed that VG-based e-cig aerosols amplified alterations in lung function, including effects on tissue damping, when compared to PG-based e-cig aerosols [54]. Studies in human patients have identified immediate differences in lung function parameters from short-term ENDS exposure, but long-term effects have not been evaluated [55]. One possibility suggested by these results is that the impact of e-cig aerosol exposure on lung function may be time-dependent and flavor specific.

French vanilla e-liquid contains three main flavoring chemicals: ethyl vanillin (at a concentration of 8.4 mg/mL), vanillin (at a concentration of 6.1 mg/mL), and maltol (at a concentration of 4.1 mg/mL) [56,57]. This results in a high e-liquid cumulative total flavoring chemical concentration of 18.6 mg/mL. While maltol is an alcohol, ethyl vanillin and vanillin are aldehydes, a chemical family that has respiratory tract irritation properties [56]. It was previously demonstrated that the cytotoxicity of ethyl vanillin, vanillin, and maltol is dose-dependent in human bronchial epithelial cells (BEAS-2B) and in mouse neural stem cells, and that these flavoring chemicals are often found at cytotoxic concentrations (>1 mg/mL) in e-liquid formulations [58]. Moreover, flavoring chemicals in e-liquids transfer readily to e-cig aerosols [59]. There also may be additional toxicity from the flavoring chemicals when they interact with the e-liquid humectants, PG and VG, as the chemical reactions that occur when they are heated and aerosolized through an e-cig device, can lead to the potential formation of aldehydes and acetals [34,58]. As mentioned previously, vanillin PG acetal is more pro-inflammatory than vanillin [27,34]. In our study, we would expect to have higher levels of vanillin VG acetals than vanilla PG acetals due to the VG/PG ratio that we used (70/30). Furthermore, the inhalation toxicity of vanillin is well established, as evidenced by the National Institute of Occupational Safety and Health (NIOSH) 8 h occupational exposure limit for vanillin by inhalation, which is 10 mg/m^3^ [34,56]. The average e-liquid daily consumption rate is about 3–5 mL for e-cig users [56,58]. Thus, the elevated cumulative ethyl vanillin and vanillin concentrations found in e-liquids, plus the daily quantity of e-liquid used, in relation to the vanillin inhalation exposure limit, fuel concerns related to the potential respiratory effects of prolonged vanilla-flavored e-cig use. In addition, the median lethal concentration (LC_50_) of vanillin by route of inhalation in mice is 41.7 mg/kg [60]. In our study, which used a representative e-cig user vaping topography profile, the mice were exposed to 240 puffs per day. We observed that the 6 week exposure to the vanilla-flavored e-cig aerosol produced distinctive effects in terms of decline lung function, increased percentage of NK cells, as well as BALF IgG1 levels (Figure 1, Figure 3 and Figure 6), when compared to the baseline e-cig aerosol composed solely of VG/PG. Overall, the well documented inhalation toxicity of vanilla and that of its base chemical flavoring constituents (ethyl vanillin, vanillin, and maltol), support our data that indicate an enhanced toxicity of the VG/PG plus vanilla e-cig aerosol compared to that of the VG/PG e-cig aerosol.

In terms of lung immune cells, alveolar macrophage numbers were evaluated by counting macrophages on H&E stained lung slides and by flow cytometry (Figure 2). Neither assessment found a significant difference with VG/PG alone or VG/PG plus vanilla. The result from H&E staining is consistent with a prior 3 day exposure study in which no differences were noted in alveolar morphology when lungs from air-exposed and ENDS-exposed mice were compared [13]. These results are also consistent with a study that found no difference in the degree of macrophage infiltration in BALF from ENDS vehicle-treated mice after 4-weeks of ENDS exposure [15]. We did, however, identify an increase in the percentage of natural killer cells in mice exposed to VG/PG plus vanilla (Figure 3C). Studies exploring the effects of e-cig use on NK cells are limited, but one study found a decrease in NK cell function with cinnamaldehyde exposure in vitro [61]. Further studies are needed to determine whether vanilla flavoring impairs NK cell function as well. While not specifically linked to e-cigarette use, NK cells have been demonstrated to play a role in the development of allergic airway inflammation and asthma [62,63]. We also identified increases in the percentage of lung cells identified as dendritic cells, CD4+ T cells, and CD19+ B cells, suggesting that a skewed VG/PG ratio can induce a humoral immune response (Figure 3D). Due to this finding, we decided to further explore a possible induction of the humoral immune response by measuring serum and BALF immunoglobulin levels by ELISA. IgG1 and IgG2b were chosen due to their ability to reduce airway inflammation [64,65]. A previous study involving traditional cigarettes identified IgG1 increases and IgG2 decreases in the serum and BALF from smokers as compared to non-smokers [66]. In the present study, VG/PG alone did not significantly increase IgG2b levels in either serum or BALF (Figure 6C–D), but there was an increased trend in IgG1 levels (Figure 6A,B). The addition of vanilla flavoring significantly increased IgG1 levels above those of the air controls. Interestingly, there was a decreased trend in IgG2b with vanilla flavoring that almost coincides with the decrease in lung function. In the literature, IgG2b levels have been positively associated with lung function and negatively with asthma [66].

Contrary to the results by Madison et al. [9], we did not see a difference in gene expression of *Sfpa*, *Sfpb*, *Sfpc*, or *Sfpd* in the lungs of mice exposed to ENDS vehicles (Figure 5). This may be explained by the different durations of e-cig aerosol exposures: 6 weeks in our study versus 16 weeks in Madison et al. [9]. Furthermore, our analysis was performed on lung tissue samples and not in BALF, and their study utilized a greater PG/VG ratio [9]. We also evaluated several genes related to immunotoxicity. We found that the e-cig aerosol composed solely of VG/PG dysregulated more genes than did the e-cig aerosol containing vanilla flavoring, 8 genes versus 3, respectively (Figure 5A). The majority of the genes altered by the exposure to the VG/PG e-cig aerosol were down-regulated (Figure 5A) and were related to metabolism of cellular protein, as well as to levels of both phospholipids and lipids (*Il-6*, *F2*, *Snca*, and *Aldh8a1*) as per Ingenuity Pathway Analysis (Figure 5B). These results could suggest a possible mechanism for the lungs to control an aberrant lipid profile. Moreover, cytokine gene expression identified an increase in *Il-6* from VG/PG e-cig aerosol exposures (Figure 5A). It was previously demonstrated *in vitro* that *Il-6* expression correlated with reduced production of pulmonary surfactant protein A (SPA) [36]. This result is also consistent with other studies [13,14,15] but is in contrast to Madison et al. [9]. This could be attributed to differences between studies in the e-liquid constituents and the VG/PG ratios used, as this ratio affects aldehydes formation in the aerosol and very few studies use the same source of e-liquid. It is worth noting that there are no e-liquid or ENDS device standards for ENDS-related health research. In the limited number of genes that we investigated, while the VG/PG e-cig aerosol did not dysregulate genes associated with oxidative stress, the VG/PG plus vanilla aerosol up-regulated the expression of *Hpx*, a biomarker of oxidative damage (Figure 5A). In addition, there was no overlap in the genes that were dysregulated by the two e-cig aerosols (Figure 5A), suggesting that flavor may contribute to the induction of molecular changes via distinct mechanisms. In our study, the flavor-specific toxicity of e-cig aerosol, without nicotine, is supported by the distinct molecular changes (Figure 5A), impaired lung function (Figure 1), increased in NK immune cells (Figure 3), and the IgG1 levels found in the BALF (Figure 6) of VG/PG plus vanilla exposed-mice compared to mice exposed solely to the VG/PG e-cig aerosols. Overall, our data suggest that when heated and aerosolized through an e-cig device, PG and VG may induce molecular changes related to lipid metabolism in the lungs. Endocannabinoids, such as 2-AG, are derived from the phospholipid membrane [67]. These lipid-based immune mediators are believed to be primarily anti-inflammatory, due to engagement with the cannabinoid receptor-1 or cannabinoid receptor-2 [68,69,70,71,72]. Alternatively, 2-AG may be metabolized by COX-2 into other anti-or pro-inflammatory eicosanoids [73,74,75]. To our knowledge, no other ENDS study has evaluated the levels of endocannabinoids following exposure to ENDS or ENDS vehicle. Here, we demonstrated an increase in 2-AG levels following exposure to VG/PG, independent of vanilla (Figure 4A). Furthermore, we identified an increase in 12-HETE (Figure 4F) and an increased trend in PGE2 (Figure 4D), PGD2 (Figure 4E), and AA (Figure 4G), all eicosanoids, or in the case of AA, a polyunsaturated omega-6 fatty acid derived from 2-AG metabolism which have been shown to elicit both pro-inflammatory [76,77,78,79] and anti-inflammatory effects [80,81,82,83]. While it is unclear what role these compounds play in immune responses following ENDS exposure, we have identified an additional mechanism in which ENDS can disrupt lung lipid homeostasis.

Overall, our study shows that 6 week of inhalation exposure to VG-rich e-cig aerosols (70%/30% VG/PG), without nicotine or flavoring, increases the populations of immune lung cells, as evidenced by the percentage of DC, CD4 and CD19 cells (Figure 3), as well as the extracted amounts of endocannabinoid (2AG) and eicosanoid (12-HETE) in the lungs of mice (Figure 4). This e-cig aerosol exposure also dysregulated the expression of eight genes related to immunotoxicity, with functional networks associated with metabolism of cellular protein and lipid homeostasis (Figure 5). In contrast, the 70%/30% VG/PG plus vanilla e-cig aerosol exposure affects lung function (Figure 1), increases the percentage of NK, DC, CD4 and CD19 immune lung cells (Figure 3), as well as the amount of 2AG and 12-HETE in the lungs of mice (Figure 4). This exposure also dysregulated the expression of 3 genes, including up-regulation of *Hpx*, which is associated with oxidative stress (Figure 5). The BALF levels of IgG1 also were significantly elevated in this exposure group (Figure 6). Taken together, our results suggest that exposures to only e-cig delivery vehicles VG/PG, without nicotine, affect the lungs, and that addition of vanilla flavoring may enhance the lung responses.

## 4. Materials and Methods

### 4.1. Chemicals and Reagents

Fluorescence antibodies used for flow cytometry and some antibodies used for ELISA were purchased from Biolegend (San Diego, CA, USA). Authentic standards of oleoylethanolamine-d4 (OEA-d4), palmitoylethanolamide-d4 (PEA-d4), 2-arachidonylglycerol-d8 (2-AG-d8), anandamide-d8 (AEA-d8), arachidonic acid-d8 (AA-d8), and prostaglandin E_2_-d4 (PGE2-d4) were from Cayman Chemical (Ann Arbor, MI, USA). Liquid chromatography-mass spectrometry (LC-MS/MS) solvents (LC-MS grade) were purchased from Thermo Fisher (Waltham, MA, USA).

### 4.2. Mice

The 6-week old C57BL/6 female mice were obtained from Jackson laboratories (Bar Harbor, ME USA). We selected to conduct this study on female mice since previous epidemiological studies reported increased e-cig use (>50%) among women [84,85,86]. Mice were shipped to Louisiana State University and acclimated for 2 weeks before initiation of study. Subgroups of mice were randomly distributed into their respective groups: (1) high-efficiency particulate air (HEPA)-filtered air, (2) 70%30% VG/PG, and (3) 70%30% VG/PG + French vanilla flavoring (vanilla) (*n* = 11–12 per group). Mice were housed and handled in accord with the National Institutes of Health (NIH) Guide for the Care and Use of Laboratory Animals. All procedures and protocols were approved by the Louisiana State University Institutional Animal Care and Use Committee (IACUC) (protocol #17-095, approval date 15 November 2017).

### 4.3. E-Cig Aerosol Exposures

E-cig aerosols or HEPA-filtered air exposures were conducted in 5-L whole-body exposure chambers (Scireq, Montreal, QC, Canada) as described in Noël et al. [87]. We used e-liquids composed of 70% vegetable glycerin (VG) and 30% propylene glycol (PG), with and without French vanilla flavoring (purchased online from EC Blend; Medford, OR, USA). These e-liquids were analyzed independently by Bureau Veritas (Buffalo, NY, USA) using gas and liquid chromatography (GC LC) techniques: no nicotine was detected, and the PG concentrations were 28.1% for the VG/PG e-liquid and 19.5% for that of the VG/PG e-liquid with French vanilla flavoring. The e-liquids were aerosolized by a Scireq^®^ 3rd-Gen e-cig generator with the atomizer’s resistance and battery voltage set at 1.5 Ω and 4.1 V, respectively. Vaping was conducted under a topography profile of 3 s puff duration, and a 55-mL puff volume every 30 s. E-cig aerosols were sampled in the airstream exiting the chamber at a flow rate of 1 L/min throughout the experiment, in a cassette holding a 25 mm hydrophilic glass fiber filter with a 0.7 µm pore size (AP4002500, Millipore Sigma; Burlington, MA, USA) in order to determine total particulate matter (TPM) concentration by gravimetric analysis. The TPM concentration was also monitored continuously in real-time via a MicroDustPro (Casella; Buffalo, NY, USA). The average TPM levels in the e-cig aerosol exposure chamber were 0.041 mg/puff ± 0.03 (standard error of the mean) (total mass collected onto the filters ~9.95 ± 1.2 mg) for the VG/PG e-liquid and 0.035 mg/puff ± 0.03 for the VG/PG + vanilla e-liquid (total mass collected onto the filters ~8.31 ± 1.1 mg) (Table 1). Mice were exposed for 2 h a day, 7 days a week, for 6 weeks. At the end of the exposure period, the lung function of six mice from each group was assessed via the flexiVent^®^ system (Scireq; Montreal, QC, Canada). The remaining mice in each group (*n* = 5–6) were sacrificed by intraperitoneal injection of Beuthanasia-D (Merck Animal Health; Kenilworth, NJ, USA) and the blood as well as the lungs were collected.

### 4.4. Pulmonary Function Testing

Pulmonary function testing in mice was assessed via two techniques. (1) The day before sacrifice, whole-body plethysmography was assessed using a Buxco System (Buxco, Troy, NY, USA) as previously described [88]. We placed the mice (*n* = 8 per group) into individual chambers of the whole-body plethysmograph and we measured tidal and minute volumes as the mice were challenged with increasing doses of aerosolized methacholine (0 to 50 mg/mL). Lung responses were recorded for readings taken over a 5 min period.

(2) Pulmonary function of mice was measured on the day of sacrifice via the flexiVent system as described [88]. Prior to performing the flexiVent procedure, the mice were anesthetized by a subcutaneous injection of approximately 0.1 mL per 10 g of body weight of a ketamine—xylazine cocktail (100 mg/kg ketamine and 5 mg/kg xylazine). Briefly, mice (*n* = 6 per group) were anesthetized, tracheostomized, and connected to the forced oscillation measurements flexiVent system. Lung mechanics were determined using pre-defined scripts from the flexiVent system, for (1) lung mechanics and (2) dose-response, which was used with incremental doses of methacholine (0, 12.5 and 25 mg/mL). The single frequency forced oscillation technique (FOT) was used to measure respiratory system resistance (Rrs), compliance (C), and elastance (E), while broadband FOT was used to assess Newtonian resistance (Rn), tissue damping (G), and tissue elastance (H). Measurements were accepted only if the coefficient of determination was >0.95, assuring the fit of the single compartment model and the constant phase model. For each parameter evaluated (Rrs, C, E, Rn, G, and H), at least five measurements were averaged. Following the lung function procedure, mice were euthanized by an intraperitoneal injection of Beuthanasia-D.

### 4.5. Tissue Staining

The left lung lobe from the non-flexiVented mice was inflated and fixed in 10% normal buffered formalin (10% NBF). Fixed lung was processed and embedded in paraffin using standard histologic techniques. Paraffin embedded lung was cut into 5 micrometer sections and stained hematoxylin and eosin on an automatic stainer (Leica). Stained sections of lung were examined under a microscope and five random 400× fields, including tissues from upper, middle, and lower lung were evaluated for the number of macrophages and then normalized to number of macrophages per alveolus. Images of alveoli taken at 400× were examined for additional pathology by a board-certified veterinary pathologist. Mouse heads from non-flexiVented mice were fixed in 10% NBF and decalcified in Kristensen’s Decal Solution (50% mixture of 1N sodium formate and 8N formic acid). Fixed decalcified tissue was then processed and embedded in paraffin using standard histologic techniques. Paraffin embedded tissues were cut to 5 µm sections on a microtome. Sections were placed on a glass slide and stained with hematoxylin and eosin on an automatic stainer. Images of two random 400× nasal mucosal fields were examined by a board-certified pathologist.

### 4.6. Lung Immunophenotype by Flow Cytometry

The right inferior lung lobe of the non-flexiVented mice was homogenized in 1× phosphate-buffered saline (PBS) and an aliquot was stained via one of two staining protocols. Adaptive immune cells were stained with extracellular antibodies for CD4 (PECy5), CD8 (PECy7), and CD19 (BV650). Innate immune cells were stained with extracellular antibodies for CD49b (FITC), F4/80 (PECy7), CD11b (APC), CD11c (PE), and Gr1 (PacBlue). Cells were fixed following extracellular staining and analyzed on an ACEA Novocyte Flow Cytometer. Compensation was set using antibody capture beads, and gates were set using fluorescence minus one control containing cells from each set of lungs. Innate immune cells were identified as natural killer cells (CD49+, CD11b−), alveolar macrophages (F4/80+, CD11c+, CD11b−), interstitial macrophages (F4/80+, CD11b+, Gr1−), dendritic cells (CD11b+, CD11c+), or neutrophils (CD11b+, CD11c−, Gr1+), by percent of parent immune cells gated [89,90]. Adaptive immune cells were identified by percent of parent lymphocytes as T Helper Cells (CD4+), Cytotoxic T Cells (CD8+), or B Cells (CD19+).

### 4.7. Extraction of Lipid Mediators

The right middle lung lobe from each non-flexiVented mouse was rinsed in 1× PBS, weighed, and homogenized in 3 mL of cold 2:1 (*v*/*v*) methanol:water using a Tissuemizer probe. Fifty µL of 1% (*w*/*v*) triphenylphosphine (Sigma, St. Louis, MO, USA) dissolved in methanol was added to the samples to prevent auto-oxidation of lipids. A mixture of deuterated internal standards was then added to each homogenate: AA-d8, PGE2-d4, and 2-AG-d8 (13.3 pmol each) and PEA-d4, OEA-d4, and AEA-d8 (100 pmol each). After vortexing, the samples were chilled for an hour at −80 °C. The samples were then centrifuged for 20 min at 3000× *g* at 4 °C. The supernatants were mixed with 2 mL of 1:1 (*v*/*v*) hexane:ethyl acetate (containing 0.1% acetic acid) and 1 mL of high-performance liquid chromatography (HPLC) water, while the resulting pellets were dissolved overnight at 50 °C in 3 mL of 0.1 M NaOH for protein determination. After vigorous vortexing, the samples were centrifuged at room temperature at 800× *g* for 5 min to separate the two layers. The top organic layer was transferred into a clean glass tube and the bottom aqueous layer was subjected to another round of organic solvent extraction. The pooled organic fractions were dried under nitrogen gas. One-hundred µL of methanol was used to reconstitute the samples, which were then transferred to HPLC vials for analysis by LC-MS/MS as previously described [91]. The chromatographic peak area of each lipid mediator was divided by the peak area of its linked internal standard, followed by subsequent normalization to the protein content of each lung sample. The quantities of each lipid mediator in the control lungs (no VG/PG, no vanilla exposure) were set to 1.0.

### 4.8. Lung mRNA Extraction and Gene Expression Analysis by Quantitative RT-PCR

Total RNA was isolated from the right superior lung lobe of each non-flexiVented mouse according to directions from the manufacturer, using the RNeasy Plus Mini Kit (Qiagen; Germantown, MD, USA). Quantification was performed using a NanoDrop ND-1000 spectrophotometer (Thermo Scientific; Wilmington, DE, USA). A RevertAid First Strand cDNA synthesis kit (Thermo Scientific) was used to synthesize cDNA, following the manufacturer’s protocol. Real-time PCR was performed with SYBR Green PCR Master Mix (Qiagen) on a Stratagene Mx3005P thermocycler following the manufacturer’s program recommendations. The comparative cycle threshold (ΔΔ*C*T) was used to determine relative gene expression, with GAPDH as the reference gene. Results are reported as fold change in test samples compared to control [(2^−ΔΔ*C*T^)].

### 4.9. RT^2^ Profiler PCR Array

The lungs of the non-flexiVented mice were analyzed for the expression of 84 genes related to immunotoxicity (PAMM-179Z) on an RT^2^ PCR array (Qiagen), per the manufacturer’s instructions. As previously described [92], total RNA (0.5 μg) was reverse-transcribed with the RT^2^ First Strand Kit (Qiagen 330401), and the cDNA was diluted with RNase-free water. The cDNA sample was mixed with RT^2^ SYBR Green qPCR Master mix (Qiagen 330503). A total of 25 μL of aliquots were added to the wells of the PCR Array plate containing the pre-dispensed gene-specific primer sets. We performed the PCR per the cycling conditions of the Applied Biosystems model 7300 real-time cyclers. Gene expression and fold change was calculated using the ΔΔ*C*t method, with the web-based PCR Array data analysis software. Δ*C*t data were calculated using the average geometric mean of the following genes: *Hsp90ab1*, *Gusb*, *Actb*, and *B2m*, as the normalization factor (*n* = 4 per group).

### 4.10. Immunoglobulin Level Determination by ELISA

IgG1 was measured in serum (1:100,000 *v*/*v*) and bronchoalveolar lavage fluid (BALF; 1:100 *v*/*v*) from the *flexiVented* mice following previously described procedures [93] with an anti-mouse IgG1 and biotinylated anti-mouse IgG1 antibody (Biolegend). Samples were normalized using an IgG1 standard (Thermo Fisher). IgG2b was measured in serum (1:10,000 *v*/*v*) and BALF (1:100 *v*/*v*) following the manufacturer’s protocol, using a mouse IgG2b ELISA kit (Thermo Fisher).

### 4.11. Protein Analysis for ELISA and Lipid Mediator Extraction Standardization

Serum samples were diluted 1:50 *v*/*v* in deionized water, while BALF samples and dissolved lung pellets remained undiluted (25 µL total volume) for protein quantification following the manufacturer’s instructions using a Pierce^TM^ BCA Protein Assay Kit (Thermo Fisher, Waltham, MA, USA).

### 4.12. Ingenuity Pathway Analysis (IPA)

As previously described [91,94], RT-PCR and RT^2^ Profiler gene expression data were analyzed through the use of Ingenuity Pathway Analysis (Qiagen, Ingenuity Systems, Redwood City, CA, USA) [95]. The Diseases and Functions Analysis identified the biological functions and/or diseases that were most significant from the data set. Molecules from the dataset that met the > +/−1.5 fold-change cutoff and were associated with biological functions and/or diseases in the Ingenuity Knowledge Base were considered for the analysis.

### 4.13. Statistical Methods

A two-way or one-way analysis of variance was used to evaluate differences using GraphPad Prism Software (Version 7, San Diego, CA, USA) or SigmaPlot Software (Version 11.0, San Jose, CA, USA). Grubb’s outlier test was performed to identify outliers in treatment groups. Gene expression results are considered significant with a fold-change > +/−1.5 compared to respective air control group. Significance was considered using a *p*-value of *p* < 0.05. Data from flow cytometry were analyzed after log-transformation.

## 5. Conclusions

In conclusion, this study provides more evidence that ENDS use is not benign. It is unknown what role the changes seen in this study play in pulmonary immune responses to a pathogen or to the development of chronic lung diseases, including asthma and emphysema. Our findings, however, demonstrate that ENDS delivery vehicles alone can influence markers of lung inflammation, lipid-based immune mediators, and the lung immunophenotype. Our study focused on a 70/30% mixture of VG/PG and one flavoring, French vanilla, and this is only one of thousands of possible ENDS mixtures. Overall, our study suggests that while PG and VG are GRAS food additives and may be harmless when ingested, they pose hazards to the lungs when inhaled from an ENDS device. With the increased trend in youth and non-smokers using ENDS, it is imperative that further research is conducted to tease out the complexities in the effects of ENDS on human pulmonary health.

## Figures and Tables

**Table 1:** Characterization of e-cig aerosol exposures.

	HEPA-Filtered Air	70% VG/30% PG	70% VG/30% PG + Vanilla
Temperature (°C) (± SD) ^†^	25.5 ± 1.2	26.4 ± 4.3	24.6 ± 1.6
Relative humidity (%RH) (± SD) ^†^	69.7 ± 9.3	68.0 ± 3.1	73.1 ± 4.2
Total particulate matter (TPM) concentration (mg/puff) (± SD)	---	0.041 ± 0.031	0.035 ± 0.028

**^†^** The temperature and relative humidity were measured inside the exposure chambers using a small hydrometer.

## Abbreviations

12-HETE	12-hydroxyeicosatetraenoic acid
2AG	2-arachidonoylglycerol
AA	arachidonic acid
AM	alveolar macrophages
BALF	broncho-alveolar lavage fluid
CD4	cluster of differentiation 4
CD8	cluster of differentiation 8
CD19	cluster of differentiation 19
CDC	Center for Disease Control and Prevention
DC	dendritic cells
e-cig	electronic-cigarette
COPD	chronic obstructive pulmonary disease
ENDS	electronic-nicotine delivery systems
EVALI	e-cigarette or vaping product use-associated acute lung injury
FDA	Food and Drug Administration
GRAS	generally recognized as safe
IgG	immunoglobulin G
IM	interstitial macrophages
MAO	monoamine oxidase
NEU	neutrophils
NIOSH	National Institute of Occupational Safety and Health
NK	natural killer cells
OEA	oleoylethanolamide
PEA	palmitoylethanolamide
PGD2	prostaglandin D_2_
PGE2	prostaglandin E_2_
PG	propylene glycol
VG	vegetable glycerin
SP-A-D	surfactant protein A-D
va	vanilla
THC	tetrahydrocannabinol
